# Functional capacity evaluations in medico-legal contexts: a scoping review of best practices, limitations, and areas for improvement

**DOI:** 10.3389/fresc.2026.1777330

**Published:** 2026-07-22

**Authors:** Lisa Schwab, Dominique Van de Velde, Lutgart Braeckman, Stijn De Baets

**Affiliations:** 1 Faculty of Medicine and Pharmacy, Vrije Universiteit Brussel, Brussels, Belgium; 2 Faculty of Medicine and Healthcare Sciences, Department of Rehabilitation Sciences, Occupational Therapy Research Group, Ghent University, Ghent, Belgium; 3 Faculty of Medicine and Healthcare Sciences, Department of Public Health and Primary Care, Ghent University, Ghent, Belgium

**Keywords:** disability assessment, FCE, occupational health, return to work, work capacity evaluation

## Abstract

**Introduction:**

Functional Capacity Evaluations (FCEs) are commonly used to assess individuals' capacities in relation to work demands, with growing application in medico-legal contexts. Despite their broad use, various concerns persist regarding their administration, interpretation, and application within this setting.

**Objectives:**

This scoping review synthesized evidence on best practices, limitations, and areas for improvement and innovation in the medico-legal use of FCEs.

**Methods:**

The review followed standard methodological frameworks for scoping reviews and PRISMA-ScR guidelines, searching six major databases for peer-reviewed articles published between 1990 and 2026. Studies were included if they addressed the use of FCEs in return-to-work, insurance, disability assessment, or legal contexts. Data were extracted using a standardized form and synthesized thematically across three domains: best practices, key limitations, and improvement opportunities.

**Results:**

Fifty-one articles were included. Best practices emphasized standardization and psychometrics, effort monitoring, psychosocial integration, job-specific relevance, practical feasibility, and clear reporting. Limitations included inconsistent protocols, limited validation, insufficient psychosocial integration, and concerns regarding bias and legal defensibility. Recommendations emphasized the development of modular, job-specific, and resource-sensitive protocols; stronger psychometric validation; improved evaluator training; and the establishment of interdisciplinary reporting standards aligned with international frameworks.

**Conclusions:**

FCEs hold promise in medico-legal contexts, but variability and limited psychosocial integration undermine their defensibility. Progress requires both technical refinements, including standardized, validated protocols, and a shift toward biopsychosocial, person-centered approaches that reflect real work ability.

**Systematic Review Registration:**

https://doi.org/10.17605/OSF.IO/TYEKR.

## 1 Introduction

Experts recommend the use of the International Classification of Functioning, Disability and Health (ICF) as a framework for Functional Capacity Evaluations (FCEs) (1). While a universally accepted conceptual definition of work capacity remains elusive, FCEs are conventionally defined as structured assessments designed to evaluate an individual's capacity to meet the demands of work, while considering the person's body functions and structures, environmental factors, personal factors and health status (1, 2). These evaluations typically consist of a battery of standardized performance tests simulating work activities (e.g., lifting, carrying, reaching, and hand dexterity) to determine physical workload demands and safe performance levels. In this context, “safe performance levels” are defined as the maximum level an individual can sustain prior to overexertion or risk of injury (3). FCEs are widely used in clinical rehabilitation and occupational health by providing objective data to support work-related decision-making, including return-to-work (RTW) planning, job placement, and the development of personalized rehabilitation strategies. In addition to their clinical relevance, FCE results are also frequently used in insurance and compensation contexts, making their accuracy and fairness essential to both individual outcomes and system-level decisions. This relevance extends across diverse medical contexts (e.g., orthopedics, cardiology) and occupational groups, where FCE outcomes may determine employability, from physically demanding blue-collar work to less physically intensive white-collar roles (4, 5).

Despite this widespread use, a significant challenge remains regarding the considerable variation in how FCEs are administered and interpreted. This variability undermines their reliability in guiding RTW decisions and may contribute to differences in recommendations. Differences in evaluator training, testing protocols, equipment, and administration environment contribute to inconsistency in outcomes and reduce comparability across evaluations (6–10). Furthermore, concerns about evaluator bias, the influence of compensation contexts, and the limited integration of psychosocial factors have further raised questions regarding defensibility and interpretive validity, particularly in medico-legal contexts where decisions carry legal and financial consequences (11, 12).

In response to these challenges, several authors have emphasized the need for more tailored and context-sensitive FCE practice. Standardized physical assessments alone may insufficiently capture the complexity of real work capacity, which is shaped by job demands (13), psychosocial context (14), workplace conditions (15) and individual lived experience. A growing body of work suggests that FCEs may be most meaningful when embedded within a biopsychosocial framework that contextualizes physical results and aligns evaluation content with the individual's functional profile and vocational goals (16, 17).

Moreover, there is a growing concern in the literature that FCEs are still too often interpreted in isolation, with insufficient integration of psychosocial, occupational, or environmental factors that shape functional performance (16). Such oversights are particularly problematic in medico-legal settings, where cross-national differences in legal standards and evidentiary requirements lead to marked variability in how FCE findings are used (18, 19).

While several studies have investigated the clinical applicability and psychometric properties of FCE protocols (20, 21), there remains a need for greater clarity and consensus regarding their use in medico-legal contexts. Unlike clinical rehabilitation, evaluations in these settings pose unique challenges, as results directly influence eligibility for compensation, insurance coverage, or disability benefits. A comprehensive synthesis of these issues is needed to guide interdisciplinary practice and distinguish medico-legal requirements from general clinical utility. Optimizing FCE use in this specific domain may ultimately support more equitable and effective rehabilitation outcomes by better aligning job demands with worker capacities (5).

To address this need, this review synthesizes current literature on FCEs, with a focus on their applications within medico-legal settings. It is guided by the following research questions: (1) “*What best practices are reported for conducting and interpreting FCEs in medico-legal decision-making contexts?”,* (2) “*What methodological and contextual limitations persist in current medico-legal FCE practice?”,* and (3) “*What opportunities exist for improvement and future research to strengthen the medico-legal use of FCEs?”.* By addressing these questions, the review seeks to inform evidence-based FCE practice that is not only clinically valid and functionally relevant, but also supportive of individualized and sustainable RTW trajectories. In doing so, it aims to support all stakeholders in making fair and defensible decisions grounded in current evidence and functional reasoning.

## 2 Methods

A scoping review methodology was selected for this study given the heterogeneity of study designs in the field, including opinion papers and descriptive studies which are particularly relevant in medico-legal contexts but often excluded from systematic reviews. This approach is particularly suited to mapping the breadth of available evidence and identifying conceptual, research and application gaps. Consequently, the review follows the methodological framework proposed by Arksey and O'Malley (22) which comprises the following stages: identifying the research question, identifying relevant studies, selecting studies, charting the data, and collating, summarizing, and reporting the results. The review is reported using the guidelines of the Preferred Reporting Items for Systematic Reviews and Meta-Analyses (PRISMA, see Supplementary Appendix S1) extension for Scoping Reviews (23) and the protocol was pre-registered on the Open Science Framework (https://osf.io/tyekr).

For the purposes of this review, the medico-legal context is defined broadly to encompass any setting where FCE results are used for adjudicative purposes. This includes civil litigation, workers' compensation claims, and administrative proceedings within social security or private insurance systems where functional data directly determines benefit eligibility or work capacity status. It is worth noting that definitions of disability vary across the jurisdictions and legal frameworks represented in the included literature; this review does not attempt to resolve these definitional differences but acknowledges them as an inherent feature of the medico-legal landscape.

### 2.1 Search strategy

A comprehensive literature search was conducted in PubMed, Scopus, Web of Science, APA PsycInfo, Embase and ERIS (accessed via EBSCOhost) in June 2026. The search targeted articles published from January 1990 until June 2026, corresponding to the period in which FCE emerged as a distinct, formalized assessment method (24). Both free-text terms and database-specific indexing terms were used, with Boolean operators and truncation applied as appropriate. The search strategy centered on the term “Functional Capacity Evaluation”, which is the predominant terminology used in the literature to describe this assessment approach. This term was selected to maintain high specificity for formalized assessment batteries; pilot searches using synonymous terms (e.g., work capacity assessment) were found to yield a high volume of general clinical literature unrelated to formalized FCE protocols. The medico-legal context was ensured through manual screening of titles and abstracts during the selection phase rather than through restrictive database-specific legal keywords. Publication date limits were applied, and database-specific syntax was adapted as appropriate. Grey literature was not included, and no manual hand search was conducted. The full search strategies for all databases are provided in Supplementary Appendix S2.

### 2.2 Eligibility criteria

To ensure methodological rigor appropriate for a scoping review, eligibility criteria were defined using the Population, Concept, and Context (PCC) framework recommended by the Joanna Briggs Institute for Scoping Reviews (25). The considered population was defined as working adults (typically aged 18–67 years), the investigated concept represented FCEs, and the context encompassed medico-legal, RTW, insurance, or disability assessment settings. To maintain the review's focus, RTW contexts were included only if the FCE results were used for adjudicative or compensatory purposes.

Based on the PCC framework, studies were eligible for inclusion if they were peer-reviewed articles, reviews, or guidelines published in English, German, Dutch or French. Study protocols, editorials, conference abstracts, and case reports were excluded. Research focusing exclusively on pediatric populations or describing FCEs used solely for clinical rehabilitation without discussion of medico-legal relevance was also excluded. Eligibility criteria are summarized in Table 1.

**Table 1:** Operational Definitions for Eligibility Criteria (PCC Framework).

**Categories**	**Inclusion Criteria**	**Exclusion Criteria (Reason to Exclude)**
Population	Working-age adults (18–67 years) undergoing FCE.	Children (0–18); older adults (67+) as seldom part of working population. (Wrong population)
Concept	FCE as primary outcome in methodological practices (standardization, validity, reliability, bias), interpretation, or application.	Studies that did not utilize or report results from a formalized FCE battery; purely technical/medical data; surgical/pharmacological efficacy. (Wrong outcome)
Context	Medico-legal contexts (e.g., insurance, disability assessment, RTW with adjudicative implications).	Purely clinical rehab planning without legal/insurance link. (Wrong context)
Study Design	Peer-reviewed published articles or books, including expert opinion/text papers.	Study protocols; editorials; case reports; conference abstracts. (Wrong publication)
Language	English, French, German, and Dutch.	All other languages. (Wrong language)
Timeframe	Published after 1990 (last 35 years) to ensure relevance to current practice.	Published before 1990. (Wrong study date)

### 2.3 Study selection

**Figure 1:**
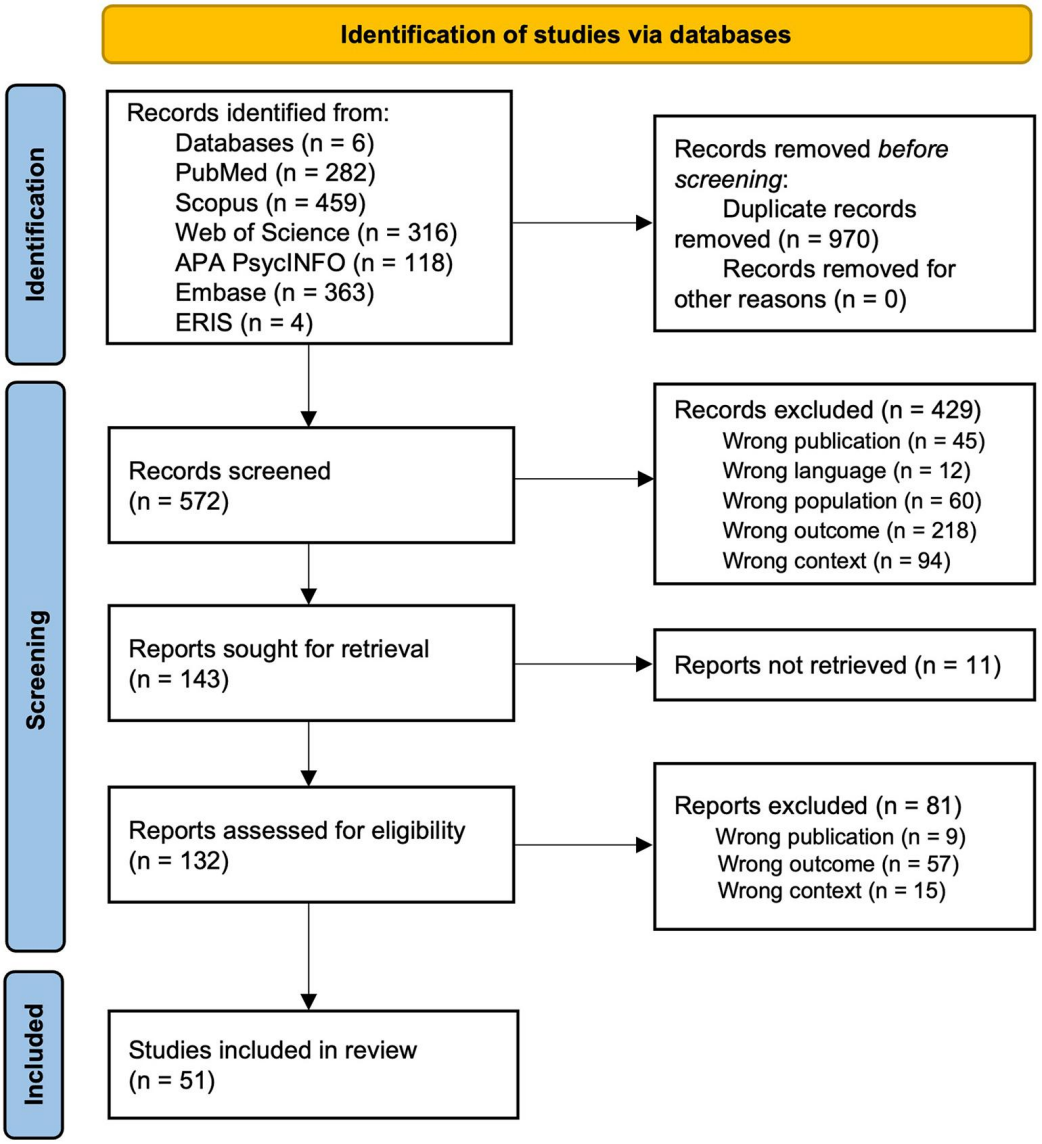
PRISMA flowchart of study selection process. Adapted from “PRISMA 2020 flow diagram template for systematic reviews” by Page *et al.*, licensed under CC BY 4.0.

All identified references were imported into Rayyan (26), and duplicates were removed. Two reviewers (L.S., S.D.B.) independently screened titles and abstracts for eligibility, while remaining blinded to each other's decisions to minimize selection bias. Inter-rater reliability for both title/abstract and full text screening was quantified using percentage agreement and Cohen's Kappa (*κ*), as it accounts for agreement occurring by chance (27). Full texts of potentially eligible studies were then reviewed against the inclusion criteria. Following the established scoping review framework (22), any disagreements were resolved through iterative consensus discussion between the two reviewers; if a consensus could not be reached, a third senior researcher was available for consultation to ensure a final decision. The study selection process was documented using a PRISMA flow diagram (see Figure 1).

### 2.4 Data extraction

A standardized data extraction form, developed in Microsoft Excel and inspired by the Cochrane data extraction form (28), was used by two researchers (L.S., S.D.B.) to capture key information. This included author(s) and year, country of study, type of FCE system used, context of application (e.g., medical, insurance, legal), identified strengths in FCE administration and interpretation, recommendations for best practices and areas of improvement. Data extraction was performed systematically by the first author (L.S.) and independently checked by a senior researcher (S.D.B.) to ensure accuracy and consistency, with discrepancies resolved through discussion and consultation with a third reviewer.

### 2.5 Data synthesis

The extracted data were synthesized using a descriptive and thematic approach, consistent with stage 5 of the Arksey and O'Malley framework (22). To ensure a structured analysis, findings were grouped and compared across three key areas, which served as the primary analytic framework: (1) best practices for conducting and interpreting FCEs in medico-legal contexts, (2) current gaps and limitations in FCE practice, and (3) areas for improvement in FCE administration. Categories were identified through iterative reading and comparison of the extracted findings. Initial inductive coding was performed by the primary researcher (L.S.) to identify recurrent patterns and concepts. These codes were subsequently cross-checked and verified by a senior researcher (S.D.B.) to ensure the reliability and comprehensiveness of the thematic structure. Any discrepancies in categorization or naming were resolved through iterative consensus meetings. This process involved interpreting and categorizing the raw data into the overarching thematic domains presented in the results, rather than utilizing verbatim reporting. The findings were summarized both narratively and in tabular form to highlight recurring categories. The allocation of findings to each category was based on interpretive synthesis, guided by how authors framed their findings rather than by formal evidence hierarchies. It should be noted that these three analytical categories are not mutually exclusive; the same study may contribute to more than one section depending on how its findings were framed by the authors.

### 2.6 Critical appraisal

To enhance the validity and transparency of this review, a risk of bias (RoB) assessment was conducted to evaluate the methodological quality of the included studies. The Joanna Briggs Institute (JBI) Critical Appraisal Tools corresponding to each study design were used to assess methodological quality (29). For mixed-methods studies, the Mixed Methods Appraisal Tool (MMAT) was applied (30). Each item was scored as yes, no, or unclear/not applicable following tool guidance. RoB ratings were performed by one reviewer (L.S.), and a random sample of 10 articles (20% of the included studies) was independently checked by a second reviewer (S.D.B.), with discrepancies addressed by consensus. The critical appraisal was conducted not to exclude studies, but to characterize the evidentiary strength of the field. Given the shortage of randomized controlled trials in medico-legal research, identifying the prevalence of high-bias descriptive studies is crucial for interpreting the reliability of current best practices.

### 2.7 Reporting results

The results are presented in alignment with the three key focus areas outlined in the synthesis process, with summary tables providing an overview of study characteristics, extracted outcomes, and the frequency of recurring categories. The narrative description elaborates on these patterns, highlighting methodological differences and thematic consistencies across studies.

## 3 Results

### 3.1 Study selection

A total of 1,542 potentially relevant records were identified through searches across six databases: PubMed (*n* = 282), Scopus (*n* = 459), Web of Science (*n* = 316), PsycInfo (*n* = 118), Embase (*n* = 363), and ERIS (*n* = 4). After removing 970 duplicates, 572 unique records were screened by title and abstract. Of these, 429 were excluded for reasons including wrong publication type (*n* = 45), language (*n* = 12), population (*n* = 60), outcome (*n* = 218), or context (*n* = 94). Title and abstract screening was conducted independently by two reviewers (L.S., S.D.B.), with 91.8% agreement and a Cohen's *κ* of 0.79 (95% CI 0.73–0.85), reflecting substantial agreement (27, 31). 47 initial conflicts were resolved through consensus discussion. Full-text screening was performed for 143 articles, of which 92 were excluded due to wrong publication type (*n* = 9), inaccessibility (*n* = 11), wrong outcome (*n* = 57), or context (*n* = 15). Reviewer agreement for full-text screening was 97.2% (*κ* = 0.94, 95% CI 0.88–1.00), reflecting almost perfect agreement, with four conflicts resolved by consensus. Ultimately, 51 studies were included in the final review. The study selection process is summarized in Figure 1.

### 3.2 Critical appraisal

Critical appraisal ratings for all 51 included studies are presented in Supplementary Appendix S3. Cross-sectional studies (*n* = 12) were generally well-described, though most failed to identify or address confounding factors. The case series (*n* = 1) showed limitations typical of its design, including absent inclusion criteria and incomplete participant selection, with uncertainty regarding the consecutiveness and validity of case identification. Cohort studies (*n* = 4) showed adequate outcome measurement, though two did not consistently report follow-up completeness. The prevalence studies (*n* = 2) showed the weakest overall profiles, with limitations in sampling, sample size justification, and response rates. The qualitative study (*n* = 1) showed gaps in researcher positionality and transparency. Randomized and quasi-experimental studies (*n* = 5) confirmed appropriate randomization or causal clarity, but blinding and participant comparability were frequently inadequate or unreported. Systematic reviews (*n* = 4) demonstrated clear research questions and appropriate search strategies, but none assessed publication bias. Textual and opinion-based papers (*n* = 21) generally met criteria for expertise and engagement with the literature; approximately half did not acknowledge limitations of the presented argument. The mixed-methods study (*n* = 1) showed no clear methodological failures but uncertainty across several criteria, including sample representativeness, nonresponse bias, and the integration of divergent findings.

### 3.3 Best practices in conducting and interpreting FCEs for medico-legal purposes

Best practices for the administration and interpretation of FCEs were clustered into six categories: (1) standardization and psychometrics, (2) effort and motivation in assessment, (3) psychosocial and contextual factors, (4) job matching and functional relevance, (5) practical considerations, (6) reporting and communication. In this context, best practices represent established, evidence-based methods and consensus benchmarks reported in the literature as the current standard for valid FCE administration. Each category is described below with supporting evidence from the included studies (see Table 2).

**Table 2:** Integrated overview of the best practices, limitations, and recommendations for medico-legal FCE use.

**Categories**	**Current Best Practices**	**Medico-Legal Limitations**	**Recommendations for Improvement**
Standardization and Psychometrics	Use of standardized, validated protocols with calibrated equipment (21, 24, 33, 34, 36, 37) Protocol-specific evaluator training and regular calibration across populations (12, 32, 35, 38, 39) Clarifying purpose of assessment, screening for contraindications and obtaining informed consent (17, 40–43). Embedding results in structured frameworks (e.g., ICF) (1, 14, 21, 44) Selection of tests with demonstrated reliability and validity, including endurance assessments, with protocols validated across populations (1, 21, 39) Use of criterion-referenced documentation, avoiding sole reliance on normative data or automated scoring outputs (32, 45).	Lack of consensus on terminology (1, 21) Absence of a “gold standard” and limited psychometric evidence (21, 63, 64) Low prognostic accuracy for RTW outcomes (21, 64, 65)	Develop modular or short-form FCE protocols to increase feasibility and reduce costs (62, 70) Adopt structured frameworks and criterion-referenced standards (e.g., ICF) (1, 14, 54) Expand psychometric validation studies and refine benchmarks (21)
Effort and Motivation in Assessment	Monitoring effort using a triangulation of validated tests, observations, and physiological measures (41, 42, 46, 47) Examiner training and blinded assessments where feasible (41) Distinction between inconsistent performance and malingering (35, 42, 47)	Difficulty distinguishing submaximal effort from person-specific functional limits (48, 50, 58) Potential for response distortion in evaluation contexts (66)	Nuanced, context-aware interpretation of invalid effort (42, 66, 71) Enhance evaluator training in effort assessment, behavioral observation and medico-legal reasoning (32, 58)
Psychosocial and Contextual Factors	Assessment of physical, cognitive, and psychosocial domains through objective and self-reported data (48–52) Interpretation in relation to each individual's unique psychosocial profile (35, 48, 51) Integration of findings with medical history and patient perspectives (40, 53, 54)	Evaluation often isolated to physical metrics, underrepresenting psychosocial and contextual factors (15, 67) Insufficient capture of environmental and social context (15, 67, 68)	Use psychosocial screening tools to contextualize findings (50, 52) Integrate results with other sources (e.g., clinical observations, patient-reported outcomes) (47, 56)
Job Matching and Functional Relevance	Tailoring tasks to specific referral questions and job demands (13, 37, 40, 45, 55–60) Using work simulations that mirror actual job activities (45, 57, 59, 60) Comparing outcomes to specific job requirements rather than population norms (11, 33, 41, 45, 61)	Poor ecological validity (35) Generic test components fail to replicate workplace reality (14) Over-reliance on broad job classifications (e.g., DOT) (45, 56)	Conduct formal job analyses and onsite job evaluations (45, 72, 73) Develop task-specific modules that simulate the critical demands of the claimant's actual job (41, 49)
Practical Considerations	Streamlining protocols to balance comprehensiveness with efficiency (33, 62) Ensuring safety via clear termination criteria (33, 46) Accounting for temporal fluctuations in capacity (13, 24, 34)	High cost and time requirements (62) Economic burden not justified by marginal increase in predictability (59, 64)	Conduct FCEs early in the disability process to improve RTW outcomes (59, 64) Consider and record time of day during testing (34)
Reporting and Communication	Transparent documentation of methods, results, and observations (13, 46, 56, 57) Narrative explanations provided alongside raw scores (13) Explicit linking of findings to functional demands and limitations (13, 56) Consistent terminology use (e.g., ICF) (14, 21) Interdisciplinary input and adherence to ethical and legal standards (40, 42)	Inconsistent documentation of clinical reasoning (56) Terminology misuse and lack of legal robustness in court or insurance proceedings (33, 42, 69) Unsystematic narrative reporting reduces comparability and transparency (44)	Use clear language understandable to all stakeholders (13, 24) Adhere to interdisciplinary standards of communication (24)

#### 3.3.1 Ensuring standardized and psychometrically sound administration

Best practices for administration emphasized the use of standardized, validated protocols with clear administration guidelines, calibrated equipment, and trained or certified evaluators (12, 21, 24, 32–39). Clarifying the purpose of the assessment, screening for contraindications to ensure safe participation, and obtaining informed consent, including cultural and language adaptations, were also stressed (17, 40–43). For interpretation, several studies recommended embedding results in structured frameworks, such as the ICF (1, 14, 21, 44).

Moreover, a strong emphasis was placed on psychometric rigor. Best practices included selecting tests with demonstrated reliability and validity, validating protocols across populations, and incorporating endurance assessments where appropriate (1, 21, 39). Transparent and criterion-referenced documentation was regarded as essential, with authors cautioning against sole reliance on normative data or automated scoring outputs (32, 45).

#### 3.3.2 Systematically assessing performance validity

Ensuring valid effort was consistently highlighted as a cornerstone of credible FCE practice. This involved the use of validated effort tests, structured observational methods, and physiological indicators, as well as examiner training and, where feasible, blinded assessments (41, 42, 46, 47). Interpretation of effort requires caution to distinguish submaximal effort from true impairment: inconsistent or reduced performance should not be equated with malingering but instead understood in the context of multiple indicators and potential psychosocial or environmental influences, which reinforces the need for personalized interpretation of effort indicators, recognizing that individual emotional, motivational, and contextual factors shape performance during FCEs (35, 42, 47).

#### 3.3.3 Integrating psychosocial and contextual information

Several studies emphasized that FCEs should be situated within a broader biopsychosocial framework. Best practices included assessing multiple domains (physical, cognitive, and psychosocial) through both objective measures and self-reported data (48–51). For example, Asante et al. (52) showed that self-efficacy beliefs were independently associated with FCE lift performance, illustrating how self-reported psychosocial measures should be assessed alongside objective tests to identify discrepancies that may signal barriers to performance. Complementing this, Oesch et al. (50) demonstrated that nonorganic somatic signs independently predicted scores across multiple FCE tasks, underscoring that observed performance cannot be interpreted in purely physical terms. Administration practices should therefore account for psychosocial influences on task performance, as behavioral and environmental factors have been identified as central determinants of FCE performance (35).

Ultimately, the interpretive value of an FCE depends on the evaluator's ability to synthesize these diverse data sources into a cohesive functional profile. Rather than reporting isolated physical scores, a defensible interpretation must contextualize performance within the individual's broader vocational and environmental reality, allowing for a transparent distinction between physiological impairment and context-dependent work disability (40, 53, 54).

#### 3.3.4 Aligning assessment content with vocational demands

Administration practices emphasized tailoring tasks to the referral question, i.e., the specific clinical or legal question posed by the referring party, and actual job demands, using simulations that mirror real work activities and aligning assessments with vocational or rehabilitation goals (13, 37, 40, 45, 55–60). This tailoring underscores the personalized nature of FCE administration, in which test content is adapted to the individuals real vocational context, thereby increasing the degree to which test conditions reflect real-world work demands (i.e., ecological validity) and relevance to RTW decisions. Outcomes were considered most useful when compared directly to specific job requirements rather than generic norms (11, 33, 41, 45, 61).

#### 3.3.5 Ensuring operational feasibility and safety

Efficiency and physical safety, i.e., the prevention of injury or harm to the assessee during testing, were recurring concerns in the included studies. Best practices included streamlining protocols to balance comprehensiveness with feasibility, avoiding unnecessary repetition, ensuring participant's physical safety with clear termination criteria, and adapting protocols to available resources (33, 46, 62). Some studies further noted that results from a single test session should not be overgeneralized, as capacity may fluctuate over time and repeated measures may provide a more accurate picture. For instance, Kyi et al. (34) found healthy adults tend to improve in performance from morning to afternoon, suggesting that late-day declines observed in FCE patients are not a normal diurnal effect and therefore warrant careful clinical interpretation. Similarly, Isernhagen (24) described a two-day testing format to determine whether the worker's status had been altered by the first day of testing itself. Strong et al. (13) further reported that injured workers raised concerns about the representativeness of single-session testing, noting that a worker's ability on the day of assessment may not reflect their functional capacity over the longer term, nor their ability to perform activities repetitively in their job.

#### 3.3.6 Providing clear and legally defensible documentation

Transparent and accessible reporting was viewed as integral to defensible FCE practice. Studies emphasized the need for clear documentation of methods, results, and observations, with findings explicitly linked to functional demands and physical safety considerations (13, 46, 56, 57). Reports were considered most useful when they included narrative explanations alongside scores, described abilities and limitations in language understandable to clinicians, employers, insurers, lawyers, courts, and the assessees themselves (13), and used consistent terminology such as ICF concepts to enhance comparability across settings (14, 21). Clear narrative reporting also enables personalized communication of an individual's functional strengths and limitations, facilitating tailored decision-making among stakeholders (13, 56). Interdisciplinary input and adherence to ethical and legal standards were also highlighted as critical for ensuring credibility and defensibility of FCE findings (40, 42).

### 3.4 Key gaps and limitations in current FCE practices

While Section 3.3 outlines established benchmarks, the following section identifies the persistent methodological and contextual barriers that prevent these standards from being consistently realized in practice. Despite the widespread implementation of FCEs, several persistent limitations and gaps in the use of FCEs were identified. Regarding standardization and psychometrics, a prominent issue remains the lack of consensus on terminology and the absence of a “gold standard” for measuring work ability (1, 21, 63). Many FCE systems demonstrate weak psychometric evidence, particularly concerning inter-rater reliability and predictive validity; some research suggests that prognostic accuracy for RTW outcomes can be as low as 10% (21, 64, 65).

A significant challenge involves effort assessment and bias mitigation. Authors note that an individual's unique psychological state can impair performance, making it difficult for evaluators to distinguish submaximal effort from person-specific functional limitations (48, 50, 58). This is exacerbated by the potential for response distortion in evaluation contexts, as moral and social motives have been shown to influence the authenticity of self-reported complaints during disability assessments (66). Furthermore, there is a lack of integration of psychosocial and contextual factors. Many FCEs rely heavily on isolated physical performance, failing to account for environmental barriers, social context or workplace reality (15, 67, 68).

Additional gaps were identified in job matching and functional relevance. Studies highlighted a lack of ecological validity, noting that generic simulated test components often fail to replicate the complex realities of an individual's workplace (14, 35). Authors noted that results based on broad job classifications [e.g., Dictionary of Occupational Titles (DOT) classifications] frequently do not reflect the variability of modern job demands or the individual's specific vocational context (45, 56). Finally, practical and reporting barriers also hinder FCE utility. Full FCE protocols are frequently described as time-consuming and costly, creating an economic burden that may not be justified by the marginal increase in predictability (59, 62, 64). Issues in reporting and communication, including inconsistent documentation and terminology misuse, frequently undermine the legal defensibility and robustness of FCE findings in insurance or court proceedings (33, 42, 56, 69). Unsystematic narrative reporting formats further reduce the comparability and transparency of functional assessments (44).

### 3.5 Recommendations for improvement in FCE practices

To bridge the gap between established benchmarks and current limitations, the following recommendations synthesize proposed innovations and future directions aimed at strengthening the future medico-legal utility of FCEs. Several areas for refinement were consistently noted. Regarding standardization and psychometrics, there is a strong call to improve feasibility and reduce costs through the development of modular or short-form FCE protocols (62, 70). Furthermore, to align with international practice, researchers emphasized the use of structured frameworks (e.g., the ICF) to harmonize FCE terminology and data (1, 14, 54). Strengthening the psychometric foundation of FCE through expanded validation studies remains a priority to ensure the reliability of results in medico-legal contexts (21).

Improvements regarding effort assessment and bias mitigation were also frequently highlighted. Rather than relying on simplistic “pass/fail” effort tests, recommendations advocate for a nuanced, context-aware interpretation of invalid effort that considers pain and moral reasoning (42, 66, 71). Evaluator training was identified as critical here, with calls to enhance education in effort assessment, behavioral observation, and medico-legal reasoning (32, 58). Additionally, to enhance interpretive validity, many studies proposed a shift toward psychosocial and contextual integration. This includes the routine use of psychosocial screening tools to assess self-efficacy and non-organic somatic components (50, 52). These findings should not be interpreted in isolation; instead, clinicians are advised to integrate FCE results with clinical observations and self-reported outcomes to support contextually informed and cautious interpretation (47, 56).

Regarding job matching and functional relevance, authors advised the integration of formal job analyses and onsite job evaluations to ensure simulated tasks accurately mirror the critical demands of the claimants' actual work environment (45, 72, 73). By tailoring the FCEs to an individual's specific job tasks and environment, authors noted that findings become directly applicable to the worker's specific vocational demands (41, 49). Finally, recommendations touched upon practical considerations and reporting. Authors highlighted the need for FCEs to be conducted early in the disability process to improve RTW outcomes (59, 64) and suggest that time of day should be recorded during testing to capture effects of circadian rhythm (34). To ensure these findings translate into action, reporting must be transparent, written in clear language understandable to all stakeholders, and adhere to interdisciplinary standards of communication (13, 24). A tabular overview of these findings is provided in Table 2.

## 4 Discussion

This review synthesized evidence on best practices, limitations, and recommendations in the administration and interpretation of FCEs with a specific focus on medico-legal contexts. Findings across the 51 included studies converged on six thematic categories: standardization and psychometrics, effort and motivation in assessment, psychosocial and contextual factors, job matching and functional relevance, practical considerations, and reporting and communication. Best practices highlighted the value of standardized protocols and psychometric rigor, multidomain and job-specific assessments, structured effort monitoring, attention to practical feasibility, and transparent reporting and communication. At the same time, persistent gaps were highlighted, including the lack of methodological consistency across tools, limited integration of psychosocial and contextual factors, practical barriers such as cost and time, and concerns regarding examiner bias, interpretive validity, and legal defensibility. To address these limitations, the review identified several actionable points: the development of modular and resource-efficient FCE designs, the expansion of psychometric validation studies, and the systematic incorporation of psychosocial screening tools. Furthermore, enhancing evaluator training and establishing interdisciplinary standards for communication are essential to ensure that FCE results are clinically valid and transparent for all stakeholders. As only a minority of the studies met all critical appraisal criteria, the synthesis highlights promising practices but should be interpreted with caution, keeping these quality concerns in mind. Together, these findings address the three objectives of this review — identifying best practices, characterizing key limitations, and proposing areas for improvement — and provide a comprehensive and structured foundation for advancing the context-sensitive and legally defensible use of FCEs in medico-legal decision-making.

This review expands on earlier concerns in the literature regarding variability in FCE practice and potential bias, issues that have been raised in prior narrative overviews and professional commentaries (33, 35), as well as in more recent systematic reviews (10, 21). However, unlike previous reviews, which often focused broadly on clinical utility or specific protocols, the current synthesis offers a clearer thematic structure and explicitly focuses on methodological inconsistencies in a medico-legal context. The methodological inconsistencies identified in this review, including the absence of a unified gold standard, variable inter-rater reliability, and lack of consensus terminology, are not merely technical limitations within individual systems. They are amplified at the international level: without harmonized FCE standards, FCE results have been shown to vary substantially across countries and societal contexts (15), meaning that the same worker's functional capacity may be assessed and interpreted differently depending on where the evaluation takes place.

These methodological inconsistencies may have particularly important implications in medico-legal settings, which differ substantially from clinical rehabilitation contexts. While protocol variability in clinical practice may primarily affect treatment planning, in legal contexts it has been described as a potential source of challenge for defensibility and consistency. When FCE protocols lack a unified gold standard, the resulting reports become susceptible to vigorous cross-examination, a situation frequently characterized in the literature as “a battle of the experts”, where the methodology itself, rather than the claimant's actual capacity, is on trial (33, 35). This review suggests that without rigorous, standardized administration, the evidentiary weight of FCE reports is diminished, which may contribute to inconsistent or contested adjudication outcomes.

### 4.1 Implications for practice and policy

The findings of this review carry distinct implications for stakeholders involved in the administration and interpretation of FCE results. For clinicians, findings related to standardization, psychometric rigor, and psychosocial integration highlight the importance of calibrated training, interdisciplinary collaboration, and contextualized interpretation. Rather than viewing FCEs as standalone determinants, multiple studies suggest situating test outcomes within the broader psychosocial and occupational context of the individual. For insurers and legal professionals, the findings on reporting and effort monitoring suggest a shift in how FCE evidence is weighed. Results should be viewed not as definitive physiological measures of disability, but as behavioral snapshots heavily influenced by context. Legal stakeholders may benefit from prioritizing FCE reports that include narrative explanations of effort (beyond binary pass/fail) and clearly link physical data to specific job demands, as these elements significantly enhance the defensibility of the findings. For policymakers, findings on protocol heterogeneity point to the need for harmonized medico-legal standards across jurisdictions to reduce inequities in how FCE results are interpreted. From the patient perspective, findings on job-specific relevance and psychosocial integration highlight the need for equitable access to evaluations that are holistic and transparent. Patients benefit most when assessments are not only technically rigorous but also person-centered and job relevant.

A notable finding of this review is the implicit attention and at the same time need for greater personalization in both the administration and interpretation of FCEs. Although FCE protocols emphasize standardization, the evidence shows that assessments are most meaningful when they consider the individual's specific job demands, psychosocial context, and lived experience. Tailoring test components to the actual work requirements and integrating contextual factors, such as motivation, coping, and workplace conditions, enhances ecological validity and may improve perceptions of procedural fairness of FCE use, particularly in medico-legal settings (74). Personalized interpretation also helps prevent the misapplication of isolated FCE scores, supports more transparent communication across stakeholders, and reduces the risk of inequitable decisions in systems where legal thresholds vary. The literature suggests that advancing FCE practice requires maintaining methodological rigor while explicitly integrating person-centered interpretation as a complement to standardized testing. Standardized approaches offer reproducibility, psychometric comparability, and legal defensibility, but risk sacrificing ecological validity when assessment content is insufficiently tailored to the individual's specific job demands and context. Person-centred approaches address this by situating evaluation within the individual's vocational and psychosocial reality, enhancing clinical meaningfulness and perceived procedural justice, though at the cost of reduced cross-assessment comparability, which represents a particular vulnerability in adversarial legal contexts.

These approaches are not mutually exclusive; the modular FCE framework discussed below represents an attempt to integrate both. Yet even within such integrative frameworks, the embedding of psychosocial and broader contextual dimensions remains limited in practice, particularly in medico-legal evaluations where institutional pressures favour objective, quantifiable metrics. Classification frameworks such as that proposed by Grotkamp et al. (75) demonstrate that personal factors (including individual life circumstances, motivational factors, and contextual influences) can be systematically documented within the ICF's biopsychosocial model to support transparent and legally defensible socio-medical assessments. Drawing on this classification, Bökel et al. (76) established expert consensus on which contextual factors are most relevant in socio-medical disability assessments, finding particularly strong agreement around attitudes, competencies and habits, and living situation. Yet a retrospective analysis of 215 socio-medical orthopedic evaluation reports found that despite this framework, nearly half of ICF environmental factors were rarely identified, with factors related to attitudes, basic skills, and support and relationships being almost entirely absent (77).

This review has several strengths. A systematic and transparent methodology was applied, drawing on the Arksey and O'Malley (22) framework for scoping reviews and reported in line with PRISMA-ScR guidelines (23). The inclusion of diverse study types spanning both clinical and medico-legal domains enabled a broad synthesis that captures methodological as well as practical insights. The thematic synthesis provided a structured overview of best practices, limitations, and areas for improvement, while the structured critical appraisal across diverse designs strengthened the interpretability of the evidence base. Nonetheless, certain limitations must be acknowledged. The inclusion of studies spanning three decades (1990–2026) implies that findings are drawn from varying legislative and social contexts. While legal definitions of disability have evolved during this period, the review suggests that core methodological challenges (e.g., the lack of a gold standard and the tension between standardization and personalization) have remained persistent across time. Additionally, definitions of disability and medical contexts vary substantially across the included studies and jurisdictions; as this review did not systematically extract or stratify findings by these variables, the synthesis reflects patterns across a heterogeneous body of literature rather than domain-specific conclusions. The review was limited to publications in English, German, Dutch, and French, which may have excluded relevant work from other regions. Potential publication bias cannot be ruled out, and some forms of grey literature, such as unpublished legal case guidelines or policy briefs, may be underrepresented despite their potential relevance to medico-legal practice. Consequently, the findings of this review may reflect scientific and clinical consensus more prominently than specific administrative or governmental mandates, and the results should be interpreted within the context of these academic and linguistic boundaries. Furthermore, a limitation of this review is the reliance on a primary search term. Although “Functional Capacity Evaluation” is the industry standard for these batteries, it is possible that by not including broader synonymous terms or specific legal keywords in the initial string, some relevant studies—particularly those from jurisdictions using alternative nomenclature—may have been omitted. Although a RoB analysis was performed to appraise methodological quality, these issues remain possible sources of bias at the evidence base level.

The findings point to several areas for future research. First, as recommended by several articles (17, 21, 35, 50, 52–56, 64, 67, 70) there is a need to develop and validate FCE instruments that integrate physical, psychological, and contextual dimensions of work capacity. Strengthening the medico-legal application of FCEs will require explicit alignment with established frameworks such as the ICF, which situates physical capacity within broader psychological, social, and environmental domains (14, 78). Second, cross-national research should be undertaken to map and compare medico-legal applications of FCEs, with the aim of harmonizing standards and identifying policy gaps. Third, future studies should explore patient-reported experiences of FCE, particularly in insurance and legal contexts where power imbalances may shape perceptions of fairness. Finally, trials are needed to evaluate the predictive validity of FCEs for long-term RTW outcomes, ensuring that assessments are not only administratively useful but also clinically meaningful.

A critical finding of this review is the tension between the legal need for standardization and the functional need for personalization. Medico-legal systems demand standardized, objective protocols and metrics to ensure comparability and fairness. However, ecological validity requires checking capacity against the specific, variable demands of a claimant's actual job, which necessitates a personalized approach. Addressing the areas for improvement identified in this review requires moving beyond incremental refinements toward a more integrative model of FCE design. Based on the patterns identified in this review, a “modular FCE framework” can be conceptualized. In this model, a standardized core battery (ensuring psychometric rigor and legal admissibility) is administered to all claimants, followed by job-specific modules that are personalized to the individual's vocational context. This conceptual approach combines standardized benchmarks with individualized, biopsychosocial elements, which could enhance both legal defensibility and ecological validity. Future research is needed to empirically evaluate its utility for supporting medico-legal decision-making.

## 5 Conclusions

Taken together, this review highlights that the role of FCEs in medico-legal contexts cannot be strengthened solely through technical refinements in test protocols. A broader shift is required toward a biopsychosocial perspective that aligns more closely with the realities of work ability and functional capacity. Such a perspective emphasizes person-centered and job-specific evaluations, acknowledges the interplay of physical, psychological, and contextual factors, and situates FCEs within a framework of equitable and defensible decision-making across jurisdictions.

## Data Availability

The original contributions presented in the study are included in the article/Supplementary Material, further inquiries can be directed to the corresponding author.
